# The Bookworld of Medicine and Science

**Published:** 1904-09-10

**Authors:** 


### THE BOOKWORLD OF MEDICINE AND SCIENCE.

London Matriculation Directory. (Cambridge: University Tutorial Series. Price 1s. net.)

Candidates for the London Matriculation examination will find this book a useful and satisfactory guide. It includes the regulations and details to be observed by intending candidates, together with the questions set at the last examination, and gives many practical suggestions, such as the best text-books to use, and so forth.

Refrigeration in the Dairy. By Loudon M. Douglas, A.M.I.C.E. (London: William Douglas and Sons, Limited. Price 2s.)

Refrigeration has now become an essential in the dairy, not only in connection with storage, but also with butter and cheese making and other processes Anyone interested in the subject will do well to peruse this book, although it will be necessary when doing so to bear in mind that only one process is seriously considered (the sulphurous anhydride), and the illustrations show the machinery produced by one particular firm; so that the book deals with only one aspect of a many-sided problem. We do not think that the carbon-dioxide and ammonia processes can be dismissed in such a summary manner as Mr. Douglas seems to indicate.

